# Extraction, purification and anti-fatigue activity of γ-aminobutyric acid from mulberry (*Morus alba* L.) leaves

**DOI:** 10.1038/srep18933

**Published:** 2016-01-08

**Authors:** Hengwen Chen, Xuanhui He, Yan Liu, Jun Li, Qingyong He, Cuiying Zhang, Benjun Wei, Ye Zhang, Jie Wang

**Affiliations:** 1 Guang’anmen Hospital, China Academy of Chinese Medicine Science, Beijing 100053, China; 2 Postdoctoral Research Station, China Academy of Chinese Medicine Science, Beijing 100700, China; 3 Department of Pharmaceutical Chemistry, Beijing Institute of Radiation Medicine, Beijing 100850, China; 4 Key Laboratory of Chinese Materia Medica, Heilongjiang University of Chinese Medicine, Ministry of Education, Harbin 150036, Heilongjiang, China

## Abstract

Mulberry (*Morus alba* L.) is a tree species of *Moraceae* widely distributed in Southern China. In the present study, the white crystal of γ-aminobutyric acid (GABA) was purified from mulberry leaves, and its bioactivity was also investigated. The main results were as follows: first, the crude GABA was extracted from mulberry leaves by using biochemical methods. Then, the crude was purified by chromatography over an S-8 macroporous resin, Sephadex G-10, and 732 cation exchange resin to yield a white crystal. Lavage administration and exposure of GABA to male NIH mice showed no adverse effects on their growth and development. In an endurance capacity test, the average loaded-swimming time of medium dose was 111.60% longer than the control (*P* < 0.01). Further investigations showed that relative to that of model control, the respective blood lactate (BL) concentrations of low- and medium-dose were 28.52% and 28.81% lower (*P* < 0.05), whereas the levels of blood urea nitrogen (BUN) were 36.83% and 40.54% lower (*P* < 0.05), and that of liver glycogen (LG) levels were 12.81% and 17.22% lower (*P* < 0.05). The results indicated that GABA has an advantage over taurine of anti-fatigue effect. These findings were indicative of the anti-fatigue activity of GABA.

Gamma-aminobutyric acid (GABA), a non-protein amino acid, widely exists in plants and animals. GABA was first discovered in 1949 in potato by Steward *et al*.^1^. Since then, numerous studies have been conducted, which showed that it not only acts as an inhibitory neurotransmitter in mammals, but also possesses multiple pharmacological effects, including blood pressure reduction, anti-cancer, anti-convulsion, antioxidant, pain reduction, sleep improvement and memory improvement^2^,^3^,^4^,^5^,^6^,^7^,^8^,^9^,^10^,^11^,^12^. In addition, GABA has been used as a nutrition supplement for juveniles to promote mental development, as well as commonly applied in the food industry as a nutritional resource for seniors^13^. GABA is therefore an ideal candidate ingredient for various medicines and personal health care products, and has attracted the interest of research scientists around the world.

Reports on the isolation and purification methods of GABA were limited. Li *et al*.^14^ introduced an enzyme transformation technology for GABA in his monograph. Wang *et al*.^15^, Wang^16^, Guan *et al*.^17^, Li *et al*.^18^ and Choi *et al*.^19^ respectively reported the isolation and purification of GABA from microorganism-fermented supernatants. However, it’s far from enough. In the present study, the crude GABA was extracted from mulberry leaves, and a biological molecular sieve was applied to generate a pure product; all the related approaches have been reported in detail. The anti-fatigue activity of GABA was also evaluated.

## Results

### Justification of ethanol subsiding

**Figure 1:**
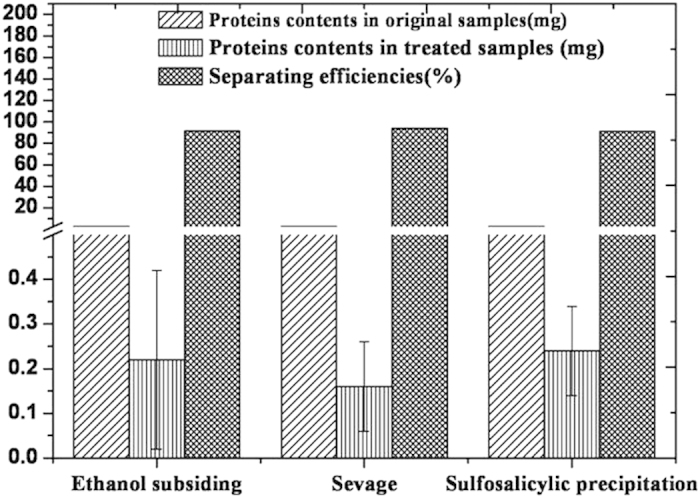
Efficiencies of different methods of separating proteins from mulberry leaves extract.

To efficiently eliminate the effects of proteins on GABA, various methods, including ethanol subsiding, sulfosalicylic precipitation and Sevage method, were compared in terms of separating proteins from mulberry leaves crude extracts. The results were expressed as the mean ± standard deviation (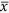 ± s) and presented in Fig. 1.

**Figure 2:**
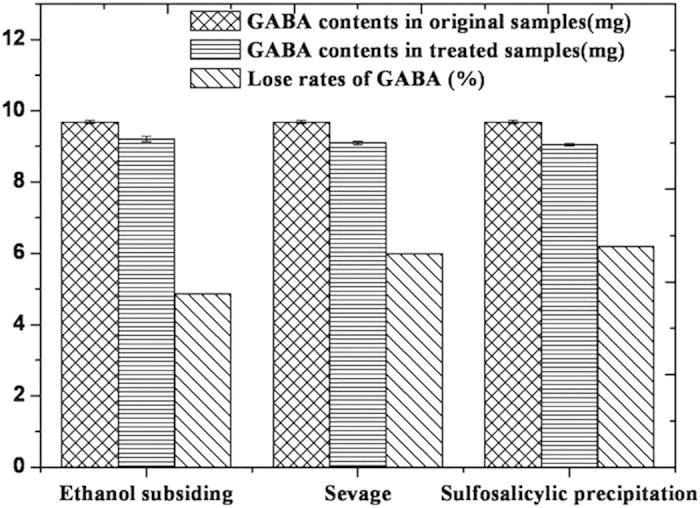
Effects of different methods on GABA.

Figure 1 showed that all three methods presented considerable efficiency (>90%) in separating proteins from mulberry leaves extracts (*P* > 0.05). Sevage method was the most efficient (efficiency rate: 93.90%), followed by ethanol subsiding (efficiency rate: 91.41%). Figure 2 showed that all those methods were associated with a loss of GABA to a certain degree (*P* > 0.05), and ethanol subsiding resulted in a 4.86% decrease in GABA content.

Ethanol subsiding was the second most efficient method in separating proteins; it was able to separate pigments, polysaccharides, tannins and other species, the remaining proteins were readily separated by gel chromatography. Moreover, it resulted in the least amount of GABA lost during procedure. Comparative analysis indicated that ethanol subsiding is the optimal method in separating proteins.

### Justification of S-8 macroporous resin

**Figure 3:**
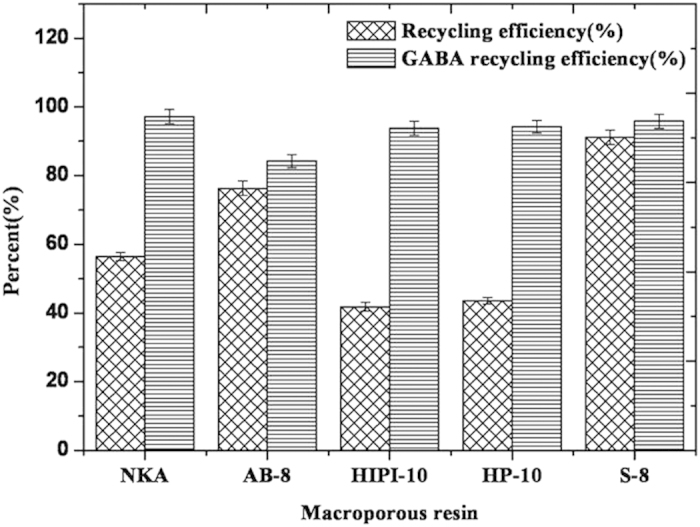
Decoloration ratio and recovery rate of 5 resin species.

The decoloration and recycling efficiencies of the five resin species were compared at 25 °C and pH 7.0 (Fig. 3). S-8 macroporous resin was superior to the other four species, with a decoloration ratio of 91.17%, whereas all the species showed no significant adsorption of GABA, and recovery rate of GABA treated on S-8 was 95.79%. Comparative analysis showed that the S-8 macroporous resin was optimal for decoloration.

**Figure 4:**
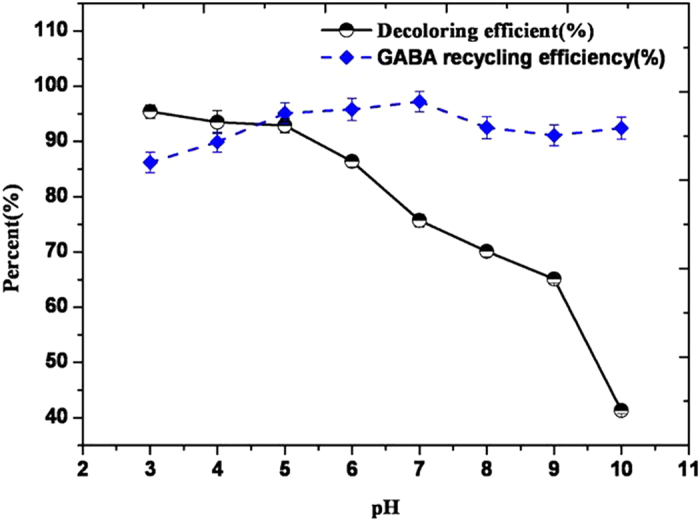
Effect of pH on decoloration ratio and recovery rate.

Effect of the pH of the loaded sample solutions on decoloring and recycling efficiencies of S-8 macroporous resin was examined under static conditions (Fig. 4). At pH < 5, S-8 showed significant decoloring efficiency, whereas recycling efficiency was relatively low. However, at pH > 5, decoloration ratio sharply dropped and a small change in recycling efficiency was observed. Comparative analysis showed that pH 5 was optimal for the loaded sample solutions and the chromatography column systems.

**Figure 5:**
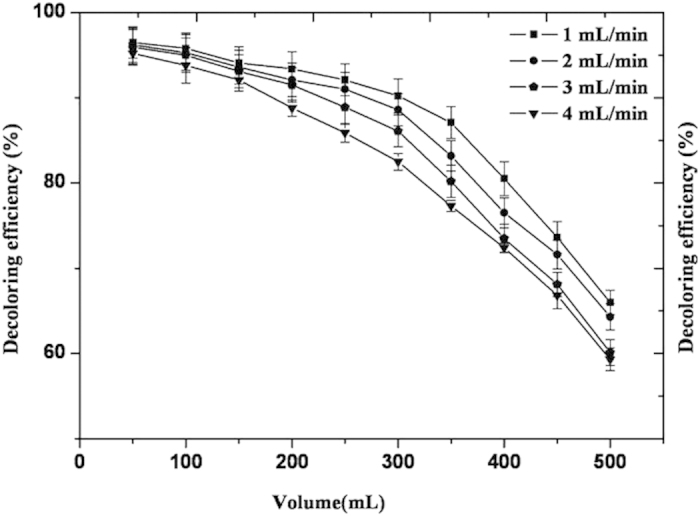
Effect of flow rate on decoloration ratio.

The effect of flow rate on dynamic adsorption action was studied by loading sample solutions onto an S-8 macroporous resin at various flow rates, namely, 1 mL/min, 2 mL/min, 3 mL/min, and 4 mL/min. The data are presented in Fig. 5. Figure 5 shows that loading sample solutions at a high flow rate can result in insufficient adsorption. On the other hand, an extremely low flow rate can lead to difficulties in operation, which in turn would increase process cost. Comparative analysis showed that the optimal flow rate was 2 mL/min. At a fixed flow rate of 2 mL/min, decoloring efficiency was decreased when the sample volume surpassed 200 mL because of the fixed amount of the silica gel and resin. Thus, 2 mL/min was determined since the assurance of the decoloring efficiency and roduction requirement. Finally, a 200-mL sample solution loaded at a flow rate of 2 mL/min was determined to be the optimal condition, which resulted in a decoloring efficiency of 92.14% and a recycling efficiency of 90.58%.

Based on these findings, the optimal conditions for S-8 macroporous resin chromatography were determined as follows: 200 mL of sample solution treated by using ethanol subsiding was acidified to pH 5, then loaded at a flow rate of 2 mL/min into a well equilibrated column packed with S-8 macroporous resin, eluted, the fractions were pooled and evaporated under vacuum.

### Purification on Sephadex G-10

**Figure 6:**
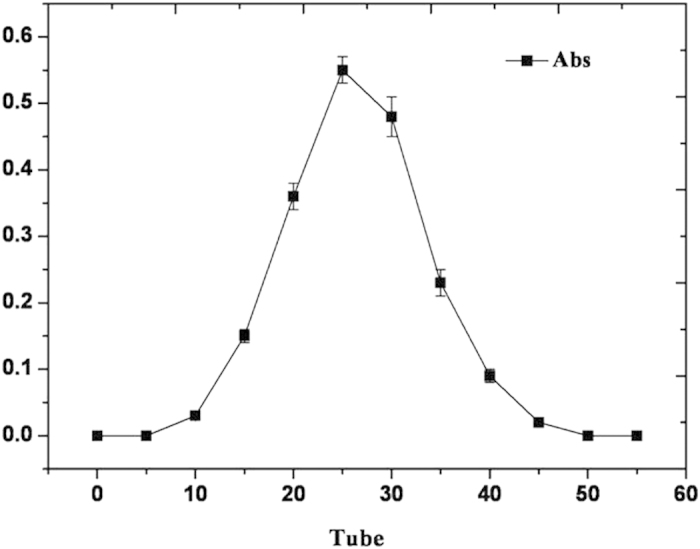
Optical absorbance of eluents separated by chromatography over Sephadex G-10.

Figure 6 showed the optical absorbance of the eluent by chromogenic reaction with ninhydrin. GABA was detected in tubes 15 to 40, and these fractions were pooled and concentrated by rotary evaporation.

### Purification on 732 cation exchange resin

The GABA crystal obtained from this step was determined to be of high purity. TLC showed the fractions of tubes 19 to 22 separated by chromatography of 732 cation exchange resin, and the red color represents a chromogenic reaction, which is indicative of the presence of GABA. The fractions containing GABA were pooled together and freeze-dried to yield GABA as a white crystal.

### Identification of GABA

Retention time: Both GABA reference and test sample were analyzed under the same chromatographic conditions, with an identical retention time of 8.591 min. Spectrum of test sample was exactly the same as that of the GABA reference, indicating that the test sample was GABA.

The GABA reference was analyzed by HPLC to construct a standard curve, from which a linear regression equation was established: A = 8083.6 × –15.7 (R = 0.9999, n = 5). Determined method has good linear relationship in the scope of 0.05 to 5.00 mg/mL. This analytic method was of high precision, with an RSD of 1.13%, and high recovery rate of 99.74%. By using this validated method, the test sample was determined with a purity of 96.32%.

### Clinical observations

No apparent item-related or toxicologically relevant changes in overall appearance, behavior, physical condition, food, or water consumption were observed in the treated animals relative to that of the normal control and model control.

**Figure 7:**
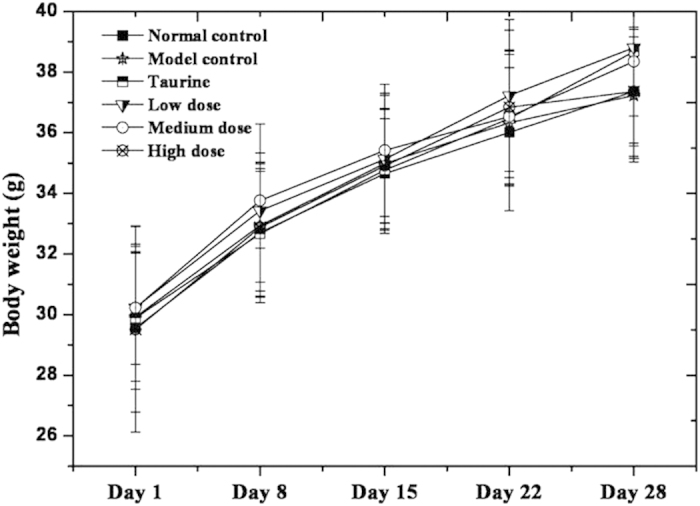
Body weights and rate increases in mice.

No apparent item-related or toxicologically relevant changes in absolute and relative body weights were observed in the treated animals compared to that of the normal control and model control. The body weights (expressed as the mean ± standard deviation) of various groups at the beginning (Day 1) and the end (Day 28) of the administration period are shown in Fig. 7. No significant differences in body weight among all groups were observed (*P* > 0.05) according to the results of the *t*-test. In general, mulberry leaf-derived GABA imparted no adverse effects on body weights.

### Endurance capacity

**Figure 8:**
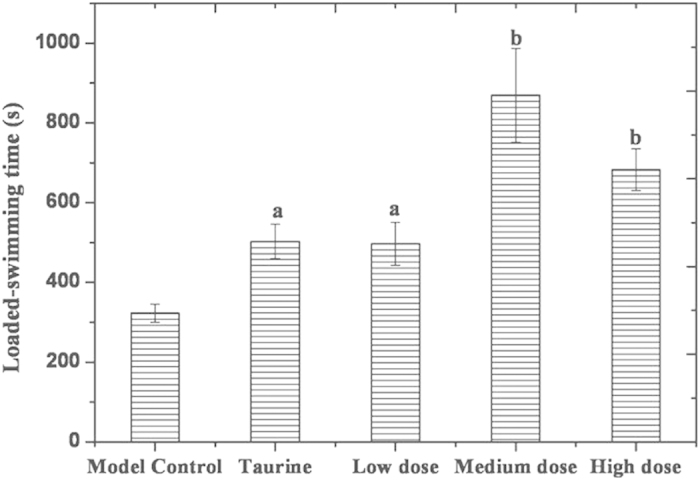
Loaded-swimming time in mice Note: the letter a represent highly significant differences (*P* < 0.01) compared to model control, the letter b indicate significant differences (P < 0.01) compared to low dose.

The loaded swimming time of treated animals was significantly longer than that of the model control, as indicated by the student’s *t*-test (*P* < 0.01). In general, comparison of loaded swimming time revealed that there were test item-related improvements in endurance capacity in the treated animals. Loaded swimming time (expressed as the mean ± standard deviation) is summarized in Fig. 8.

### Organ weights

**Figure 9:**
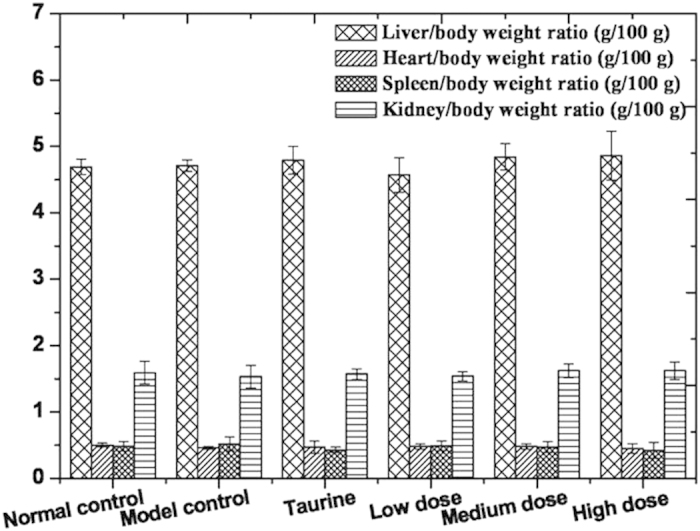
Body weights and organ/body weight ratios in mice.

The organ/body weight ratio of various treatment groups has no toxicological relevance in Fig. 9 (P > 0.05).

### Biochemical examination

Compared to that of the normal control (not treated with loaded-swimming), blood lactate (BL) and blood urea nitrogen (BUN) concentrations of the model control increased (*P* < 0.01), whereas liver glycogen (LG) concentrations sharply decreased (*P* < 0.01). These biochemical changes demonstrated that the metabolic rate of the mice was altered, resulting in more energy for physical activities such as swimming.

**Figure 10:**
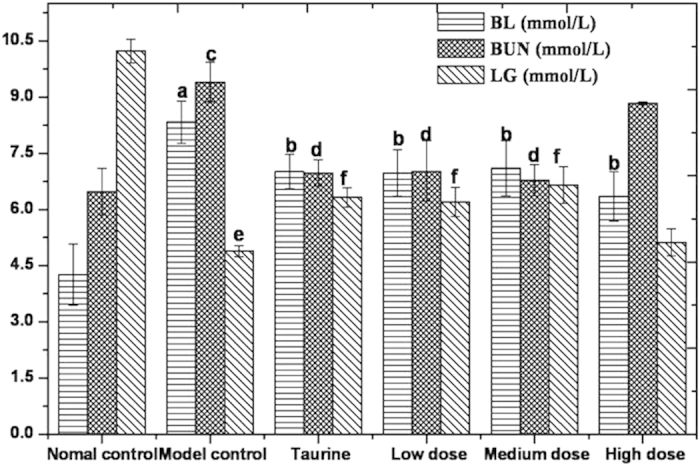
Concentrations of BL, BUN, and LG Note: Model control is compared with normal control. The letters of a, c and e represent significant differences (*P* < 0.01). Drug treated groups are compared with model control group. The letters of b, d and f represent significant differences (*P* < 0.05).

Changes in the levels various biochemical parameters were observed in the test-item treated groups relative to that observed in the model control. BL and BUN concentrations of low dose and medium dose were remarkably lower than that of the model control (*P* < 0.05). LG concentrations were significantly higher than that of the model control (*P* < 0.05). These biochemical changes demonstrated that mulberry leaf-derived GABA possessed anti-fatigue activity, which in turn improves the endurance of mice. The results of biochemical examinations of various treatment groups are summarized in Fig. 10.

## Discussion

### Isolation and purification of GABA from mulberry leaves

A biological molecular sieve is commonly used to preserve the natural activities of some biological active substances during their isolation and purification. In the present study, isolation and purification methods were determined by comparisons, and then the processing conditions were optimized to yield mulberry leaf-derived GABA with high purity.

At phase I, proteins in the mulberry leaves extract were separated through ethanol subsiding. This method has been proven to be optimal with limited cost for yielding GABA. At phase II, the S-8 macroporous resin was determined and applied to separate the pigments from the extract, and then compared with other 4 resin species in terms of decoloration efficiency and effect on GABA. The optimal conditions for S-8 macroporous resin were established as follows: no more than 200 mL of the sample solution treated by ethanol subsiding was acidified to pH 5, and then loaded at a flow rate of 2 mL/min. Under this optimal condition, the decoloration ratio and recovery rate were 92.14% and 90.58%, respectively. At phase III, pure GABA was eluted on Sephadex G-10 at a flow rate of 3 mL/min. At the last phase, 732 cation exchange resin was applied to generate highly purified GABA, and the process conditions were as follows: the sample was acidified to pH 3 and loaded at a flow rate of 2 mL/min, and it was eluted with fresh water until the pH reverted to 6, and then replaced with 0.25 mol/L ammonia water. The targeted product was obtained by freeze-drying. Macroporous resin is a type of nonionic macromolecular polymer with a macroporous structure, and commonly prepared from divinyl benzene, styrene, methyl propiolate and other materials. Macroporous resin has unique adsorption and screening properties due to its highly effective surface area, in addition to other advantages such as higher loading capacities, improved selectivity, faster adsorption speed, rapid desorption and regeneration, and insolubility in acid, alkali, and organic solution. It has been widely used in separation, desalting, enrichment, and particularly in the isolation of weak polar and non-polar products from aqueous solutions^20^,^21^,^22^. In the present study, macroporous resin was highly effective in the isolation and purification of GABA from mulberry leaves extract. When separating pigments on a S-8 macroporous resin, the sample loaded at a flow rate of >2 mL/min would cause insufficient adsorption, whereas loading at a flow rate of <2 mL/min would increase the cost of production; therefore, loading at a flow rate of 2 mL/min was determined to be optimal.

Amino acids with different numbers of acidic and basic groups will have different net charges. This can be exploited as a means of separating amino acids by using column chromatography of ion-exchange resin. Highly charged amino acids are more tightly bonded to the resin; therefore, these can be retarded in their passage through the column^23^. In the present study, GABA was eluted and purified on a column of 732 cation exchange resin by changing the pH of the elution buffer.

GABA bears nonzero net electrostatic charges at all pH values except pH = isoelectric point (PI)^24^. When pH > PI, GABA is negatively charged (an anion), whereas when pH>PI, it becomes positively charged (a cation). Consequently, in this study, samples were loaded into an ion exchange resin at pH = 3.0 ( > PI), with majority of GABA positively charged. In addition, some other external conditions may also affect GABA yield. For example, a high rate of loading sample solutions can lead to insufficient adsorption and cause wastage, whereas a low rate can prolong elution time and raise the cost of the procedure.

### Anti-fatigue activity of mulberry leaf-derived GABA

Fatigue is a state that develops a period of mental of bodily activity and characterized by a significant decrease in capacity for work and a reduction in the efficiency of accomplishment, usually accompanied by a feeling of weariness, sleepiness, or irritability^25^. Generally, endurance capacity and biochemical tests are utilized to assess fatigue. As the most convenient and objective indicator, endurance capacity is reflected by time required to perform specific activities such as climbing a rod and swimming. There are also other various biochemical indicators, including energy substances (blood glucose, liver glycogen, and muscle glycogen), metabolic regulatory substances (enzymes, hormones), and metabolic products (BL, BUN, and acetone).

Endurance capacity, together with three other biochemical indicators, BL, BUN, and LG, are sufficient in evaluating anti-fatigue activity. Endurance capacity coupled with ≥ 2 positive biochemical test results are sufficient in drawing a conclusion that the tested item possesses anti-fatigue activity^26^. In the present study, male NIH mice underwent oral administration of mulberry leaf-derived GABA for 4 weeks. The positive results of loaded-swimming time, BUN, BL, and LG were obtained from both low- and medium-dose groups, and positive results of loaded swimming time and BL were also obtained from the high-dose group. These findings indicate that both low- and medium-dose GABA improved the endurance capacity in mice. More importantly, the results have shown that GABA possessed anti-fatigue activity.

### Perspectives for mulberry leaves

GABA can currently be generated using two approaches of chemical synthesis and biological extraction^27^. Served as food and medicine, mulberry leaves from various species are rich in GABA. For example, the GABA content of *Morus atropurpurea* Roxb. is 0.862%, that of *Morus mongolica* is 0.726%, *Morus alba* L. 0.640%, *Morus multicaulis* 0.473%, and Kang Qing No. 10, 1.011%, which is the highest^28^. Mulberry trees are widely cultivated in South China’s Guang Xi and Guang Dong provinces, with a total cultivated area of 750,000 hectares, and producing 52,500–60,000 kg of mulberry leaves per hectare^29^. Consequently, mulberry leaves are explored not only as a new resource for GABA, but also for the production of silk. These studies are bound to generate highly important scientific discoveries that can also potentially improve various commercial industries.

## Materials and Methods

### Materials

Species of tested mulberry: Kang Qing *NO*.10 (E113.35, N23.12, collected from the mulberry garden of South China Agricultural University, Guangzhou, China). Taurine (H44023028, Guangzhou Baiyunshan Pharmaceutical Stock Co. Ltd., China).

### Preparation of crude GABA extracts

100 g of ground dry mulberry leaves were collected in a beaker, to which 3,400 mL distilled water (1:34, sample:water, w/w) was added. The leaves were soaked for 15 min, then extracted by using ultrasonication, followed by microwaving at 144 W for 165 s. The mixture was then centrifuged at 3,000 rpm for 10 min, the supernatant was then isolated and concentrated by rotary evaporation under vacuum until a volume of 50 mL remained.

### Separating proteins and polysaccharides from mulberry leaves extracts by subsiding method

Ethanol subsiding. A two-fold volume of anhydrous ethanol was added to 10 mL of the extract solution, vigorously stirred. After 24 h, the solution was centrifuged at 3,000 rpm for 10 min. The supernatant was collected and concentrated by rotary evaporation, until a volume of 10 mL remained.

Sevage method. 50 mL of a mixture of chloroform and butanol (5:1, v/v) was added to 10 mL of the extract, vigorously stirred. The processing method was the same as ethanol subsiding.

Sulfosalicylic acid method. An aqueous solution of 10% sulfosalicylic acid was added to 10 mL of the extract, vigorously stirred. The processing method was the same as ethanol subsiding.

Determination of protein content. The Bradford assay was used to determine the amount of protein in solution by measuring the optical absorbance at a wavelength of 595 nm^30^.

Determination of GABA content. The Berthelot assay was performed to analyze the amount of GABA in solution by measuring the optical absorbance at a wavelength of 645 nm^31^.

After comparing the three subsiding methods described above, ethanol subsiding was determined to be the best method for separating proteins from mulberry leaves extract. Details of the procedure of ethanol subsiding were as follows, a two-fold volume of absolute ethanol was added to the extract solution (2:1, v/v), vigorously stirred. After 24 h, the mixture was centrifuged at 3,000 rpm for 10 min, the supernatant was collected and concentrated under reduced pressure. Concentrated solution obtained in this step was then subjected to further purification, which is described in the next section.

### Purification by macroporous resin chromatography

Resin, pre-soaked in 95% ethanol for 24 h, was loaded into a column (length: 70 cm, i.d.: 2.5 cm), and elution with ethanol was performed at a flow rate of 3 mL/min until the eluent mixed with water (1:5, v/v) ceased to produce a white suspension solution. Elution was then performed with a large volume of distilled water until no alcohol was detectable in the eluent. The resin was soaked in 4% (m/m) HCl aqueous solution for 4 h, followed by elution with distilled water returned to pH 7, then soaked in 2% (m/m) NaOH aqueous solution for another 4 h, followed by elution with distilled water reverted to pH 7. The resin was then equilibrated.

The macroporous resin was eluted with a mixture of ethanol (70%) and NaOH (1%) until the resin showed its original color. The resin was then eluted with water until it reverted back to pH 7. 1 g of S-8, NKA, AB-8, SIPI-10, and HP-10 macroporous resin was added to 5 identical Erlenmeyer beakers, followed by 25 mL extract solution. The solutions were then mixed on a shaking table at a rotation rate of 150 rpm for 24 h at 25 °C. The optical absorbance of solution (A) from each beaker was then measured at a wavelength of 340 nm and compared with that of the original extracts solution (A_0_). Decoloration ratio was then calculated by using Equation 1, the optical absorbance of solution (A_2_) in each beaker was measured at a wavelength of 645 nm and compared with that of the original extracts solution (A_1_). The recycling efficiency of GABA was calculated by using Equation 2.


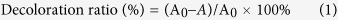


In Equation 1, A_0_ and A represented the optical absorbance of the pigment in the original extract and treated solution, respectively.


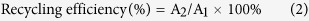


In Equation 2, A_1_ and A_2_ represented the optical absorbance of GABA in the original extract and treated solution, respectively.

The extract solution was adjusted to pH 2, 3, 4, 5, 6, 7, 8, 9 and 10, respectively. Then, 25 mL of each extract solution was poured into an Erlenmeyer beaker, followed by the addition of S-8 macroporous resin (1 g). The solutions were mixed on a shaking bed at a rotation rate of 150 rpm for 24 h at 25 °C. The optical absorbance of solution (A) in each beaker was measured at a wavelength of 340 nm and compared with that of the original extract solution (A_0_). Decoloration efficiency was calculated by using Equation 1, the optical absorbance of the solution (A_2_) in each beaker was measured at a wavelength of 645 nm and compared with that of the original extract (A_1_). Recovery rate of GABA was calculated by using Equation 2.

Determination of flow rate for loading sample solution: The different types of resin were loaded into a column (length: 30 cm, i.d.: 2.5 cm) and immersed in ethanol (loaded up to 2/3 of column height). The sample solutions were then loaded by using a constant-flow pump at a flow rate of 1 mL/min, 2 mL/min, 3 mL/min, and 4 mL/min, respectively. 50 mL of the eluent was collected from each bottle under various flow rate conditions. The optical absorbance of the collected eluent (A) was measured at a wavelength of 340 nm and decoloration ratio was calculated by using Equation 1.

The optimal purification conditions for S-8 resin were determined as follows: adjusting the concentrated solution to pH 5, then loading onto a column at a flow rate of 2 mL/min. Fractions eluted from S-8 resin column were pooled and concentrated under reduced pressure to yield a concentrated solution that was subjected to further purification.

### Purification by chromatography on Sephadex G-10

Sephadex G-10, which was pre-soaked in boiled water for 3 h to achieve extensive swelling, was mixed with water and vigorously stirred. The upper aqueous layer was then aspirated from the mixture and replaced with fresh water; this step was repeated 3 times, and left to stand to facilitate sedimentation. The concentrated solution was loaded into a column (length: 70 cm, i.d.: 2.5 cm), eluting at a flow rate of 3 mL/min with distilled water. A 9-mL eluent were collected per tube by using an auto collector. Fractions containing GABA, as detected by using the Berthelot assay or ninhydrin paper, were pooled and evaporated under reduced pressure to yield a concentrated solution that was subjected to further purification.

Berthelot assay. To save time but without compromising the test accuracy, only tubes 1, 5, 10, 15, 20, 25, 30, 35, 40, 45, 50, and 55 were analyzed.

### Purification by chromatography on 732 cation exchange resin

Pretreatment of 732 cation exchange resin: 100 g of resin was soaked in 60 °C water for 2 h with constant stirring, then filtrated under vacuum, washed with water several times, and then loaded into a column packed with distilled water at a flow rate of 2 mL/min. Elution was performed at a flow rate of 3 mL/min using 300 mL of 1 mol/L HCl in water, the resin was soaked in this acidic solution for 15 min, then eluted with fresh water until the solution reverted back to pH 7. This elution process was sequentially repeated with 300 mL of 1 mol/L NaOH and HCl in water, respectively. The resin was completely equilibrated prior to use.

The concentrated solution was adjusted to pH 3, then loaded into a column, eluting at a flow rate of 2 mL/min with fresh water until pH reverted back 6, then replaced with 0.25 mol/L ammonia water. A 6-mL eluent was collected per tube and fractions showing red in the chromogenic reaction were pooled and evaporated under vacuum. Finally, the residue was freeze-dried, yielding the desired GABA as a white crystal.

A sample of the GABA crystal was sent to the China National Analytical Center (NACC, in Guangzhou City) to determine its molecular structure and purity.

High-performance liquid chromatography (HPLC) was performed on an Agilent LC1200 equipped with a G1362A Refractive Index Detector (RID), and an analytical Hypersil ODS C18 column (length: 2.1 × 200 mm, particle size: 5 microns)., Column temperature was at 38 °C, detection wavelength was at 450 nm, the flow rate was 1.0 ml/min, sample size was 10 μL, mobile phase A: 500 mL buffer of sodium acetate 1.20 g, triethylamine 100 μL, ethylic acid 100 μL and butylene oxide 1.80 mL. Mobile phase B: 400 mL buffer of sodium acetate 1.35 g, acetonitrile 200 mL and methanol 200 mL (pH was adjusted to 7.30 by acetic acid). The mobile phase consisted of A and B was used as gradient elution (100% A to 100% B) in 0 to 20 min.

### Evaluation of anti-fatigue activity of GABA

Male NIH mice were acclimatized to the environmental conditions of the animal room for 7 days. Data recorded during the one-week pre-test period ensured that only healthy animals were selected for the study. During the whole study period, food (obtained from Southern Medical University) and water (municipal tap drinking water) were provided *ad libitum*.

The experimental animals were randomly divided into 6 groups (18 animals per group). The first two groups served as normal control and model control, and both were treated with the vehicle. The remaining groups were treated with GABA at three different doses (a low dose of 0.15 g/kg, a medium dose of 0.3 g/kg, and a high dose of 0.9 g/kg GABA/animal weight, which was 5, 10, 30 times the recommended dose for humans, respectively) and taurine dose of 0.9 g/kg GABA/animal weight. All 6 groups were treated daily by oral administration for 4 weeks, physical conditions and body weight of the animals were recorded daily prior to dose administration.

The animals were weighed daily prior to treatment. The individual dose for each animal was calculated basing on the current body weight.

After 30 min of the last administration, the experimental animals (except for normal control, 9 animals per group) were allowed to swim in a pool box (depth: 30 cm, water temperature: 25 ± 1 °C) with a piece of lead (5% body weight) attached to their tail roots. Using a stop watch, the time from initiating swimming to sinking was determined and designated as the loaded-swimming time. After swimming, all the animals were subjected to cervical dislocation.

All study animals (drowned and killed) were submitted for a complete necropsy to remove and weigh their organs (heart, liver, kidneys, and spleen). Ratio of organ weight to body weight was calculated by using Equation 3.


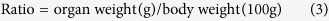


After 30 min of the last administration, the experimental animals (except for normal control, 9 animals per group) were allowed to swim in a pool box. After 90 min, all the animals were subjected to cervical dislocation. Blood samples from all study animals were collected from the orbital sinus, and the serum was separated and collected as test samples for BL and BUN analysis. Serum samples were treated with a lactate analysis kit and urea nitrogen analysis kit, following the respective manufacturer’s instructions. The concentrations of BL and BUN were calculated by using Equations 4 and 5, respectively.


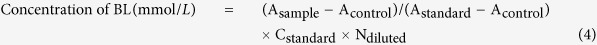



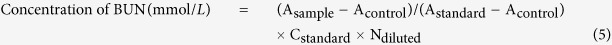


A_sample_ represents the optical absorbance of the serum sample. A_control_ represents the optical absorbance of the control sample from the kit. A_standard_ represents the optical absorbance of the standard sample from the kit. C_standard_ represents the concentration of the standard sample from the kit, 3 mmol/L and 10 mmol/L for lactate and urea nitrogen, respectively. N_diluted_ represents the dilution of each serum sample.

After 90 min swimming, liver samples from all study animals were collected and dried with filter paper. An aliquot of a 1:3 mixture of liver (mg) and alkaline solution (μL) were added to each tube, heated in boiling water for 20 min, then allowed to cool in running water. The hydrolysis solutions were further processed to yield glycogen test samples, which were measured by using a liver glycogen analysis kit, following the manufacturer’s instructions. The concentrations of liver glycogen were calculated by using Equation 6.


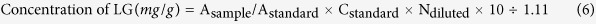


A_sample_ represents the optical absorbance of the glycogen test sample. A_standard_ represents the optical absorbance of the standard sample from the kit. N_diluted_ represents the dilution of the glycogen test sample prior to analysis.

10 was the fold dilution at measurement. 1.11 was the transfer coefficient of glucose to glycogen.

## Additional Information

**How to cite this article**: Chen, H. *et al*. Extraction, purification and anti-fatigue activity of γ-aminobutyric acid from mulberry (*Morus alba *L.) leaves. *Sci. Rep*. **6**, 18933; doi: 10.1038/srep18933 (2016).
